# Photoperiod effects on growth, lipid metabolism, and lipidomics analysis of tilapia

**DOI:** 10.3389/fphys.2026.1794531

**Published:** 2026-06-15

**Authors:** PengFei Gao, ZhanYang Tang, YuJie Cao, YuFang Cheng, Ting Huang, Bang Luo, ZhuanLing Lu, Kai Huang

**Affiliations:** 1 College of Animal Science and Technology, Guang Xi University, Nanning, China; 2 Guangxi Academy of Fishery Science, Nanning, China

**Keywords:** fatty acid profile, lipidomics analysis, liver, photoperiod, tilapia

## Abstract

**Background:**

To investigate the effects and underlying mechanisms of photoperiod on the growth and lipid metabolism of tilapia (*Oreochromis niloticus*).

**Methods:**

Four photoperiod treatment groups were established: 10L:14D, 12L:12D, 14L:10D, and 16L:8D. Various analytical methods were employed, including tissue sectioning, blood lipid analysis, gene expression profiling, fatty acid profiling, and lipidomics, to assess the impact of photoperiod on lipid metabolism in tilapia.

**Results:**

In both females and males, the highest relative body weight growth rates were observed in the 16L:8D group, reaching 122.1% and 133.6%, respectively. As the photoperiod increased, the visibility of lipid droplets in the liver decreased. The expression levels of lipid synthesis‑related genes (*fasn, acaca, srebp1*, and *aclya*) were downregulated. Blood lipid concentrations, including cholesterol (TC), triglyceride (TG), low‑density lipoprotein (LDL), and non‑esterified fatty acid (NEFA) levels, were reduced. Saturated fatty acid (SFA) content decreased, whereas polyunsaturated fatty acid (PUFA) content increased. Lipidomic analysis revealed 54 significantly differential lipid metabolites between the control group (12L:12D) and the experimental group (16L:8D) (*P*<0.05), and candidate biomarkers for photoperiod‑regulated lipid metabolism were identified.

**Conclusion:**

These findings suggest that a long photoperiod (16L:8D) effectively increases the growth rate of tilapia and reduces blood lipid concentrations and hepatic fat deposition, thereby promoting healthy growth. This study provides a theoretical foundation for healthy aquaculture practices in fish.

## 1 Introduction

Photoperiod is the phenomenon of regular alternation between light and dark periods within a day-night cycle and is the most important environmental factor that regulates circadian rhythms. Biological rhythms are regulated by highly coordinated internal and external environments to maintain normal physiological activities such as sleep, blood pressure, and metabolism. Most animals can perceive such external signals through their internal biological clocks, thereby regulating their physiological activities and behavioral patterns (Mortimer et al., 2025; Wood et al., 2020; Nisembaum et al., 2021; Qiang et al., 2021). Photoperiod is among the most critical environmental cues for organisms and plays a vital role in regulating feeding, behavior, growth, and metabolism (Lucon-Xiccato et al., 2022; Malinovskyi et al., 2022; Moran et al., 2014). Studies have shown that different seasons affect physiological states such as body weight, fat content, and metabolism in animals (Navarro-Masip et al., 2023). Changes in photoperiod may also modulate the rate of lipid metabolism, thereby influencing growth and enhancing environmental adaptability (Wang et al., 2023). Photoperiod also affects lipid metabolism in fish. For example, in the Yellow River carp (*Cyprinus carpio haematopterus*), winter photoperiod increased triglyceride (TG) levels and the expression of genes related to lipid synthesis (Wang et al., 2023). In Japanese medaka (*Oryzias latipes*), a short photoperiod reduced the expression level of the PPARα gene, thereby inhibiting lipolysis (Fujisawa et al., 2021). These findings indicate that photoperiod directly or indirectly affects lipid metabolism in fish, thus affecting their health. Therefore, emphasizing the influence of photoperiod on aquatic organisms in artificial aquaculture systems is crucial.

Lipids play important roles in various physiological functions, including substance metabolism, cell growth, and signal transduction (Watterson and Douglas, 2022). Investigating the factors that influence lipid metabolism in organisms is highly important for animal health and growth (Wang et al., 2025). Lipids are a general term for fats and lipoids, where fats refer to triglycerides (TG), and lipoids mainly include phospholipids, cholesterol, and other lipid-like substances. The main factors affecting fish lipid metabolism include the environment, energy intake, nutritional imbalance, and physiological and interspecific differences, as well as genetic and variation factors (Du., 2014). Among these, environmental factors are crucial. Photoperiod regulates the lipid metabolism rate of the Yellow River carp, thereby affecting its growth and enhancing its environmental adaptability (Wang et al., 2023). Currently, research on the effects of photoperiod on lipid metabolism in fish has focused mainly on gene transcription levels, oxidative stress, and lipid metabolism enzyme activities. A long photoperiod promoted lipogenesis, lipolysis, and fatty acid oxidation in juvenile grass carp (*Carassius auratus*) (Wei et al., 2019). Furthermore, the combined effect of a long photoperiod and temperature alters the activities of enzymes related to lipid metabolism in Japanese amberjack (*Seriola quinqueradiata*) (Fukada et al., 2023). Lipidomics is an important approach for systematically analyzing changes in the composition and expression of lipids in organisms during life processes. It enables efficient investigation of the changes and functions of lipid families and individual lipid molecules across various biological processes, thereby elucidating the underlying mechanisms of related biological activities. In fish, lipidomics has been widely used to reveal patterns of lipid metabolism changes during growth, reproduction, and environmental responses (Dreier et al., 2020). Lipidomic analysis revealed that the addition of gamma-aminobutyric acid (GABA) to the diet of yellowtail can regulate lipid components in the liver, such as triglycerides and phospholipids, thereby improving growth performance and fish meat quality (Zhang et al., 2025). Lipidomic analyses revealed clear tissue- and species-specific lipid patterns in fertilized marine fish *(Trachinotus ovatus*, *Platax teira*, *and Plectropomus leopardus*) (Liu et al., 2026). Lipidomics revealed that benzyl paraben (BzP) causes lipid metabolic disturbances, including glycerophospholipid, glyceride, and sphingomyelin disturbances, in the liver of tilapia (Lin et al., 2022). However, studies on the effects of photoperiod on lipid metabolism in tilapia are limited. Therefore, this study employs lipidomics to assess the impact of photoperiod on lipid metabolism in tilapia, and the findings are expected to fill this gap in knowledge.

Tilapia is a major aquaculture species recommended by the Food and Agriculture Organization (FAO) of the United Nations (Fu et al., 2021). Recognized for its rapid growth rate, strong adaptability, and high protein content, it has become a globally significant freshwater aquaculture species. It is also among the important freshwater farmed fish species in China, with the country’s tilapia production and export volume ranking first in the world for many consecutive years (Xiong et al., 2023). In aquaculture, it is of paramount importance to regulate environmental factors to promote lipid metabolism to achieve optimal growth rates while mitigating the risk of obesity-related diseases. Some studies have confirmed that the growth rate and gonadal development of tilapia are optimal under a 16L:8D photoperiod (Fu et al., 2021; Tang et al., 2019). However, research on the effects of photoperiod on lipid metabolism in tilapia and its underlying molecular mechanisms remains relatively scarce (Malambugi et al., 2020). With the continuous development of the aquaculture industry, the impact of environmental factors on the healthy growth of fish has become a focal point in aquaculture research. Therefore, this study investigated the effects of photoperiod on growth and lipid metabolism and the underlying mechanisms, aiming to provide a theoretical foundation for the advancement of intensive tilapia farming.

## 2 Materials and methods

### 2.1 Experimental design

The samples were Nile tilapia of the GIFT strain obtained from the Guangxi Academy of Fishery Science, Guangxi Zhuang Autonomous Region, China. This project was approved by the Ethics Committee of Guangxi Academy of Fishery Science, China (Ethics Review Number: GAFS2024006). All the experimental fish were reared and handled in strict accordance with China’s Guidelines for the Care and Use of Laboratory Animals. Before starting the experiment, the fish were acclimated in rearing tanks (200 L) for one week, with regular feeding and water changes, and were tightly wrapped with black non-woven fabric to block external light. Four different photoperiod treatments were used: 10L:14D (8:00–18:00 illumination period), 12L:12D (8:00–20:00 illumination period), 14L:10D (8:00–22:00 illumination period), and 16L:8D (8:00–24:00 illumination period). In the experiment, the photoperiod was the sole variable. To avoid any interference from gender differences, female and male fish were analyzed separately. Female fish and male fish were separately raised for the experimental study and statistical analysis. A total of 480 healthy fish (240 females and 240 males) were randomly selected and divided into 20 fish per rearing tank. There were three replicates per photoperiod treatment group. The fish were fed a diet containing 30% crude protein (Tong Wei Co., Ltd.), and the standard proportion was 2% of the daily body weight. The feeding amount of each group was kept consistent. The water in the breeding pool was replaced every three days. The light intensity was 1000 lux. The water temperature was maintained at 28 ± 1°C. The dissolved oxygen concentration was maintained above 5 mg/L. The experiment lasted for 30 days, and no deaths were recorded during the trial.

### 2.2 Sample collection

After the experiment ended, the fish were mildly anesthetized with 200 mg/L tricaine mesylate (MS-222). Six fish were randomly selected from each tank and placed in a tray filled with ice. Venous blood was collected to measure serum metabolite levels. After the fish were dissected, liver tissue samples were collected, rapidly frozen in liquid nitrogen, and stored at –80°C for various analyses, including fatty acid profiling, lipidomics, gene expression analysis, and liver histological section preparation.

### 2.3 Liver sectioning

The tilapia liver tissue was fixed in a fixation solution for 24 h. The sucrose solution was used to dehydrate the tissue, followed by rapid freezing, embedding, and sectioning (10 μm thick). Afterward, the sections were stained with oil red O for 10 min, differentiated with 60% isopropyl alcohol, and stained with hematoxylin for 5 min, after which the plates were prepared. Observations were finally made using an upright microscope (Nikon ECLIPSE C1, Tokyo, Japan). Each group consisted of 3 biological replicate samples (n=3), and each slice underwent three technical replicate experiments, resulting in a total of 72 slices.

### 2.4 Blood lipid test

Six fish were randomly selected from each of the control and experimental groups, and venous blood was collected. The blood samples were allowed to stand at 4°C for 12 h. The blood samples were subsequently centrifuged at 4000 rpm for 10 min. The serum was carefully aspirated and stored at –80°C for subsequent experiments. The serum of 2 fish was mixed in equal amounts to form a single sample, and the levels of total cholesterol (TC), triglycerides (TG), low-density lipoprotein (LDL), and non-esterified fatty acids (NEFA) were tested. For each group, equal amounts of serum from the two fish were mixed together to form a single sample, aiming to reduce individual variability. The experimental design included three replicates. The levels of these metabolites were measured using a double-antibody one-step sandwich enzyme-linked immunosorbent assay (ELISA) kit. A 96-well plate was used, and the operation was performed following the instructions of the kit: 10 μL of serum was added to each sample well to be tested, followed by 40 μL of diluent and 100 μL of horseradish peroxidase (HRP)-labeled antibody per well. The reaction wells were sealed with plate sealer and incubated in a 37°C water bath for 60 min. After the supernatant was discarded, the samples were allowed to stand for 1 min, after which the washing solution was added to each well, and this rinsing step was repeated 5 times. Substrate was added to each well, followed by incubation at 37°C for 15 min in the dark. Afterward, 50 μL of stop solution was added to each well, and the absorbance was measured within 15 min at a wavelength of 450 nm using a microplate reader (Infinite F50, Zurich, Switzerland). The experiments were carried out by Beijing Huabo Deyi Biotechnology Co., Ltd. Each group included three biological replicates, with blank controls included. Serum from 6 fish in each group was pooled to prepare 3 samples (n=6), and three technical replicates were used as blank controls.

### 2.5 Fatty acid profile analysis

Fatty acid profiles in the liver were analyzed as follows. About 50 mg of liver tissue was harvested from the median lobe of the liver. The tissue samples were saponified with a 2% sodium hydroxide-methanol solution at –80°C for 15 min, followed by methylation with 7 mL of a 15% boron trifluoride-methanol solution at 80°C for 2 min. After cooling to room temperature, 2 mL of n-heptane was added to each sample, and the mixture was vortexed vigorously for 2 min. Saturated aqueous sodium chloride solution was then added to the mixture, which was allowed to stand undisturbed for phase separation. The upper n-heptane fraction was carefully transferred to a clean test tube preloaded with 2 g of anhydrous sodium sulfate, vortexed for 1 min, and left to stand for 5 min for complete dehydration. The resulting supernatant was subsequently transferred to a gas chromatography (GC) vial for further analysis. GC analysis was performed on an Agilent 7890A GC system (Agilent Technologies Inc., Santa Clara, CA, USA) equipped with a highly polar capillary column coated with cyanopropyl polysiloxane (100 m × 0.25 mm internal diameter, 0.20 μm film thickness). The oven temperature program was optimized as follows: the initial temperature was maintained at 125°C for 2 min, increased to 180°C at a rate of 12°C/min, further increased to 200°C at 3.5°C/min, held at that temperature for 20 min, and finally increased to 230°C at 5°C/min for 5 min. The total runtime was 36 min. The injection volume was 1.0 μL with a split ratio of 10∶1, and high-purity nitrogen was used as the carrier gas. Triglyceride undecanoate (C11:0) was employed as the internal standard for quantitative calibration. Quantitative analysis was conducted by comparing the retention times of the target analytes with those of a commercial mixed standard containing 38 fatty acid derivatives. Three biological replicate samples were randomly selected from each experimental group (n=3); therefore, 24 samples were obtained for gas chromatography analysis.

### 2.6 Analysis of gene expression

Real-time fluorescence quantitative PCR (RT-qPCR) was used for quantitative analysis of multiple genes in the liver (n=3 per group). Total RNA was extracted from tilapia liver cells using TRIzol reagent (Invitrogen, Carlsbad, CA, USA) according to the manufacturer’s instructions and treated with RQ1 DNase (Promega, Madison, WI, USA) to remove DNA. The quality of the purified RNA was determined by measuring the absorbance at 260 nm and 280 nm (A260 and A280, respectively) using a SmartSpec Plus spectrophotometer (Bio-Rad Laboratories, Inc., Hercules, CA, USA). RNA integrity was further verified by 1.5% agarose gel electrophoresis. All the RNA samples were stored at –80°C for future use. First-strand cDNA was synthesized using 1 μg of total RNA as a template following the instructions of the ReverTra Ace qPCR RT Kit (TOYOBO Life Science, Shanghai, China) and stored at –20°C. The expression levels of the *srebp1*, *fasn*, *aclya*, and *acaca* genes were detected by qPCR. *β-actin* was used as the reference gene, and the primers are shown in **Table 1**. Specific primers were designed based on the cDNA sequences. qPCR was performed on a Bio-Rad S1000 using Bestar SYBR Green RT-PCR Master Mix (TOYOBO). The reaction system consisted of 20.0 μL of each forward and reverse primer, 10 μL of SYBR^®^ Premix, 1 μL of cDNA, and 8 μL of double-distilled water. The conditions included denaturation at 95°C for 1 min, followed by 40 cycles of denaturation at 95°C for 15 s and annealing and extension at 60°C for 30 s. Relative gene expression levels were calculated using the Livak and Schmittgen 2^–ΔΔCt^ method (Livak and Schmittgen., 2001). Each sample was subjected to three replicate qPCR amplifications. The specificity of the primers was verified by the melting curve, which showed a single product peak with no non-specific amplification. Agarose gel electrophoresis revealed a single target band. The primer sequences and efficiencies are shown in **Table 1**.

**Table 1:** Primers used for quantitative RT-PCR (qPCR).

Function classifications	Primer name	Sequence (5′-3′)	Eff. (%)	GenBank
Lipogenesis	*srebp1*	F: CCCCAACTTCCTTCTCTCA R: AGGCACACAACGCCATAC	98.1%	XM_005457771.4
	*fasn*	F: CTCGGGGGGTTTGGTTT R: GCTCTGTTCCTTTCAGTGTGC	95.0%	XM_003454056.5
	*aclya*	F: GGAGGTTGCCGAGGTAT R: AGGTGAGGCTGGAGATGA	102.1%	XM_025910662.1
	*acaca*	F: TCAGTCTCCCAACTCCTATG R: CCTGTCCACCTCTTCTTTC	98.1%	XM_003442027.5
Internal reference	*β-actin*	F: CACACAGTGCCCATCTACGAG R: ACGATTTCCCTCTCGGCTG	98.9%	XM_003443127.5

### 2.7 Lipidomic detection and bioinformatics analysis

We performed lipidomic analysis on the livers of tilapia from the control group (12L:12D) and the experimental group (16L:8D). Eighteen fish were randomly selected from the control and experimental groups, and their livers were collected and temporarily stored at –80°C. In the same group, the livers of 6 fish were mixed together to form one sample (n=6), with the aim of reducing the differences among different individual fish. The experimental design consisted of 3 replicate groups. The extracted lipids were separated using a UHPLC Nexera LC-30A ultrahigh-performance liquid chromatography system. Lipid analysis was conducted using a Q Exactive Plus mass spectrometer (Thermo Fisher Scientific, MA, USA). Raw data were annotated for lipids using LipidSearch software (version 4.0; Thermo Fisher Scientific). Data quality control was performed using the R package gmodels (v2.19.1) (Warnes et al., 2024). Z score normalization, orthogonal partial least squares discriminant analysis (OPLS-DA), and heatmap visualization were carried out using the R package pheatmap (v1.0.12) (Kolde and Kolde, 2015) and the R package ropls (Thevenot, 2016). The thresholds for identifying significant differences in the OPLS-DA model were a VIP ≥ 1 and a t-test *P* < 0.05.

### 2.8 Measurement and calculation of growth performance

The sample size for each group was 20 (n=20), and 3 replicate groups were established for each group. Body weight (Wt), body length (BL), body height (BH), body width (BdW), and body weight were measured at the beginning and end of the experiment. The growth performance was evaluated by calculating the relative weight gain rate, relative growth rate of the BL, relative growth rate of the BH, and relative growth rate of the BdW, which were calculated with the following formulas:


$$\text{relative growth rate of body weight}(\%)=(\text{W}{\text{t}}_{2}-\text{W}{\text{t}}_{1})/\text{W}{\text{t}}_{1}\times 100\%$$

$$\text{relative growth rate of body length}(\%)=({\text{L}}_{2}-{\text{L}}_{1})/{\text{L}}_{1}\times 100\%$$

$$\text{relative growth rate of body height}(\%)=({\text{H}}_{2}-{\text{H}}_{1})/{\text{H}}_{1}\times 100\%$$

$$\text{relative growth rate of body width}(\%)=(\text{Bd}{\text{W}}_{2}-\text{Bd}{\text{W}}_{1})/\text{Bd}{\text{W}}_{1}\times 100\%$$

In the formula, Wt_1_, BL_1_, BH_1_, and BdW_1_ are the average initial body weight, average initial body length, average initial body height, and average initial body width at the start of the experiment (g/fish), respectively, and Wt_2_, BL_2_, BH_2_, and BdW_2_ at the end of the experiment.

### 2.9 Data analysis

Owing to the significant differences between males and females in terms of growth performance and gene expression levels, these female and male data were analyzed separately to reduce experimental error. All the experimental data were processed and analyzed using SPSS 22.0. Origin 2024 and GraphPad Prism 9.5.0 (730) were used to generate graphs for data visualization. The Kolmogorov-Smirnov test and Levene test were performed to verify the normal distribution and homogeneity of variance of all the datasets. One-way analysis of variance (ANOVA) was performed on the data. Statistical significance was set at *P*< 0.05. Additionally, the Mann-Whitney U non-parametric test was performed. The experimental data are expressed as the mean ± standard error of the mean (SEM).

## 3 Results

### 3.1 Growth performance

The growth parameters of the tilapia are shown in **Tables 2**, **3**. One-way analysis of variance (ANOVA) was used to test for significant differences among the groups. In female fish, the relative growth rates of body weight, body length, and body height were the lowest at 10L:14D and the highest at 16L:8D (*P*< 0.01). There was no significant difference in the relative growth rate of body width among the groups. In male fish, the relative growth rates of body weight, body length, and body height were the lowest at 12L:12D and the highest at 16L:8D (*P*< 0.01). The relative growth rate of body width was the lowest at 12L:12D and the highest at 14L:10D (*P*< 0.01). At 16L:8D, the relative growth rates of body weight, body length, and body height were the greatest for males and females.

**Table 2:** Morphological parameter statistics of female tilapia.

Photoperiod	Body weight(%)	Body length(%)	Body height(%)	Body width(%)
10L:14D	98.7 ± 4.0^b^	22.7 ± 0.6^b^	21.7 ± 0.4^b^	25.6 ± 0.8^a^
12L:12D	108.5 ± 1.8^b^	24.7 ± 1.7^ab^	22.2 ± 1.1^b^	26.0 ± 0.7^a^
14L:10D	105.1 ± 3.6^b^	25.9 ± 0.1^ab^	23.4 ± 0.1^b^	24.5 ± 0.5^a^
16L:8D	122.1 ± 1.5^a^	27.9 ± 0.5^a^	26.1 ± 0.4^a^	26.3 ± 0.7^a^

The value for each group is presented as mean ± standard error of the mean (SEM). The sample size for each group is 20 (n=20). Different superscript letters indicate significant differences among groups (*P*< 0.05), while the same letters indicate no significant difference. These letters indicated groups difference in the upper right corner of data.

**Table 3:** Morphological parameter statistics of male tilapia.

Photoperiod	Body weight(%)	Body length(%)	Body height(%)	Body width(%)
10L:14D	107.7 ± 3.0^ab^	26.9 ± 0.7^ab^	29.9 ± 0.4^b^	33.4 ± 0.2^b^
12L:12D	93.4 ± 6.5^b^	24.2 ± 2.3^b^	24.6 ± 0.9^c^	28.9 ± 2.9^c^
14L:10D	125.5 ± 10.1^a^	30.3 ± 2.8^a^	31.6 ± 2.0^b^	41.1 ± 2.3^a^
16L:8D	133.6 ± 2.5^a^	32.2 ± 1.9^a^	36.8 ± 1.9^a^	38.2 ± 1.3^ab^

The value for each group is presented as mean ± standard error of the mean (SEM). The sample size for each group is 20 (n=20). Different superscript letters indicate significant differences among groups (*P*< 0.05), while the same letters indicate no significant difference. These letters indicated groups difference in the upper right corner of data.

### 3.2 Histological observation of the liver

**Figure 1:**
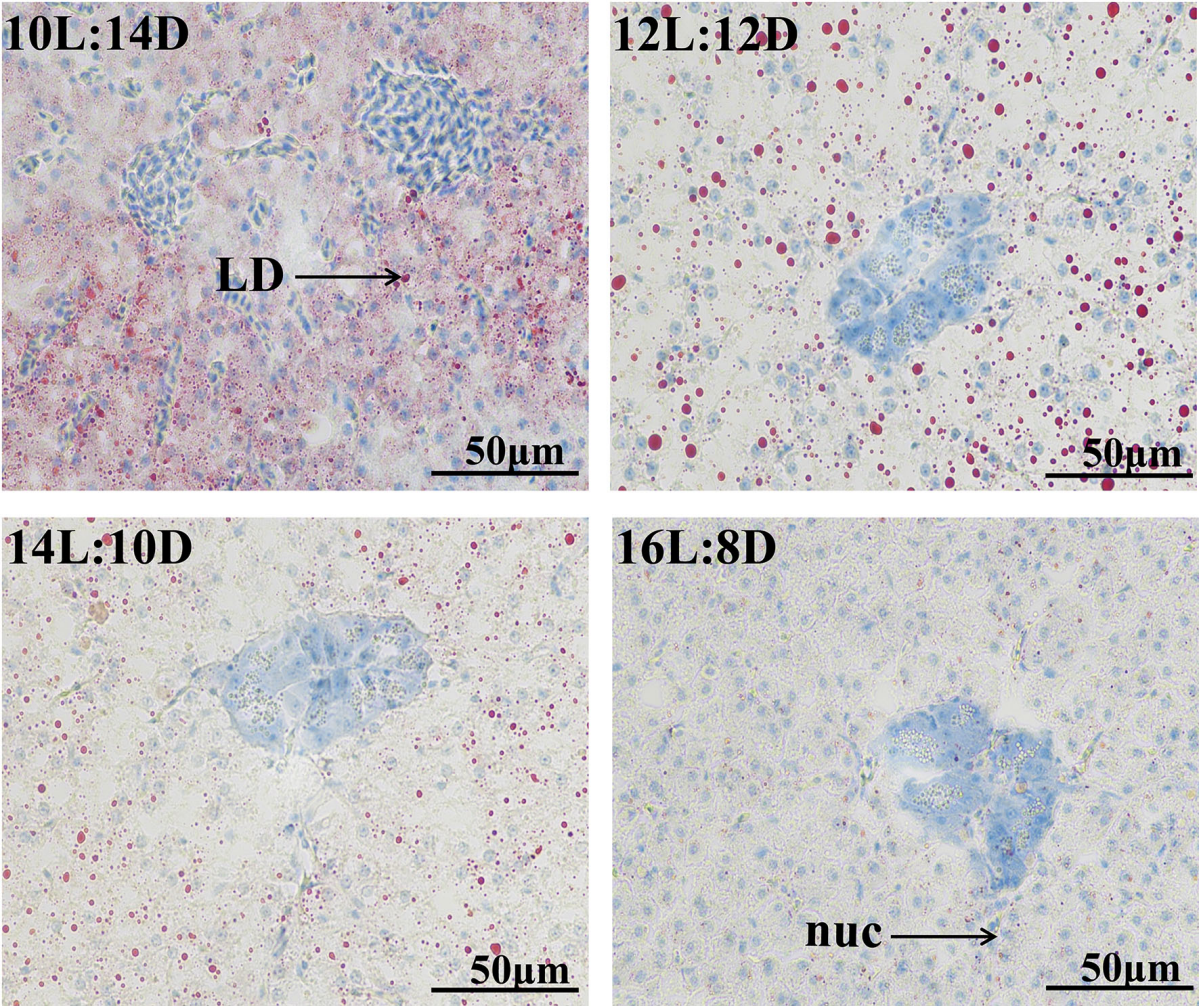
Slices of female livers. Oil Red O-stained sections of female tilapia livers under different photoperiods. Nuc is the cell nucleus. LD is a lipid drop. The scale size is 50 μm. The sample size for each group is 3 (n=3).

**Figure 2:**
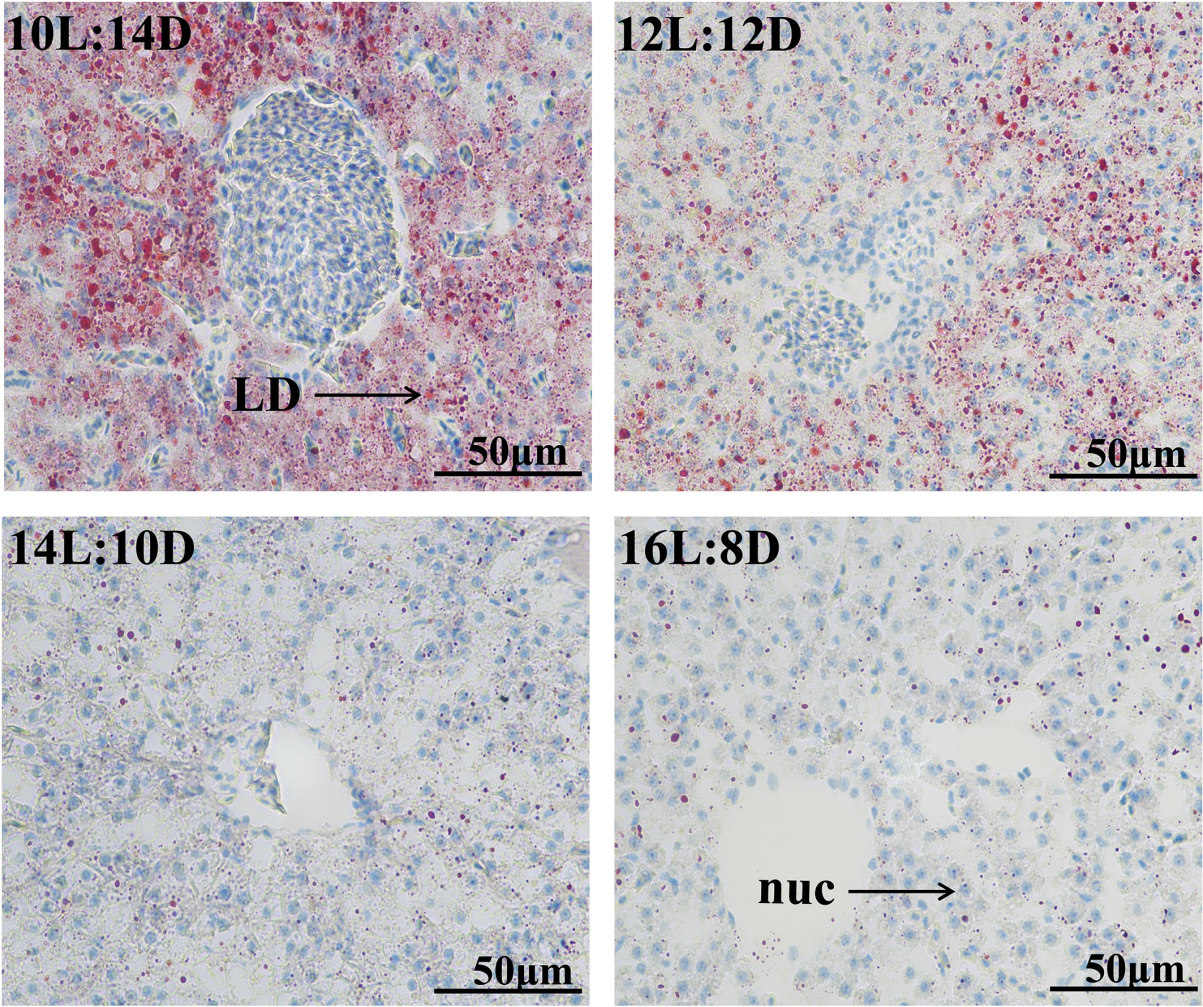
Slices of male livers. Oil Red O-stained sections of male tilapia livers under different photoperiods. Nuc is the cell nucleus. LD is a lipid drop. The scale size is 50 μm. The sample size for each group is 3 (n=3).

Liver lipid droplets have important biological functions, such as energy storage, metabolic regulation, cell protection, and signal transduction. The fewer lipid droplets there are, the less fat deposition there is. To investigate the effect of photoperiod on hepatic fat deposition in tilapia, histological observations were performed, as shown in **Figures 1**, **2**. The results revealed that as the photoperiod increased, the visibility of lipid droplets in the livers of both male and female fish gradually decreased, with the sparsest distribution observed in the 16L:8D photoperiod group.

Liver sections from female fish are shown in **Figure 1**. Under the 10L:14D photoperiod, lipid droplets were densely packed and widely distributed within hepatocytes. Under the 12L:12D photoperiod, the staining of lipid droplets became lighter, and their distribution range became more concentrated. Under the 14L:10D photoperiod, the lipid droplets became sparse and reduced in size. Under the 16L:8D photoperiod, hepatocytes displayed a normal morphology, the nuclei were stained blue, and lipid droplets were barely observable.

Liver sections from male fish are shown in **Figure 2**. Under the 10L:14D photoperiod, lipid droplets were relatively abundant and scattered in distribution. Under the 12L:12D photoperiod, the lipid droplets exhibited a regular morphology without cavitation. Under the 14L:10D photoperiod, both the size and the occurrence of lipid droplets decreased. Under the 16L:8D photoperiod, hepatocytes exhibited an intact morphology and clear nuclear staining, and almost no lipid droplets were present.

### 3.3 Blood lipid levels

**Figure 3:**
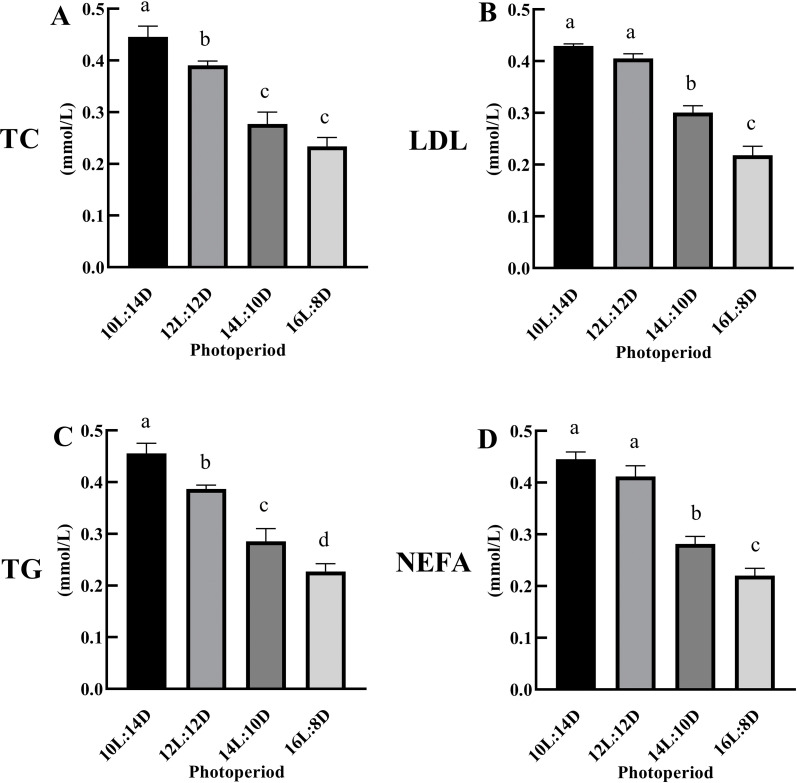
Effects of different photoperiods on blood lipids in female tilapia. A total of 4 photoperiod control groups were established. The values for each group are presented as the mean ± standard error of the mean (SEM) and were derived from 3 biological replicate groups. Each biological replicate group consisted of 3 mixed samples from 6 fish (n=6), and each sample underwent 3 technical replicate experiments. **(A)** Contents of TC in different photoperiods; **(B)** Contents of LDL lipids in different photoperiods; **(C)** Contents of TG in different photoperiods; **(D)** Contents of NEFA in different photoperiods; Different superscript letters indicate significant differences among groups (*P*< 0.05), while the same letters indicate no significant difference.

**Figure 4:**
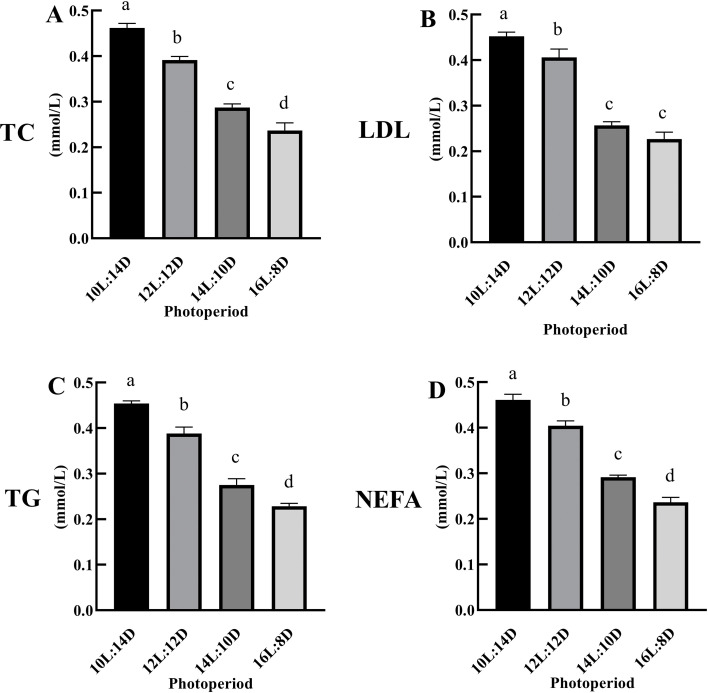
Effects of different photoperiods on blood lipids in male tilapia. TC: total cholesterol; LDL: low-density lipoprotein cholesterol; TG: triglyceride; NEFA: non-esterified fatty acid. A total of 4 photoperiod control groups were established. The values for each group are presented as the mean ± standard error of the mean (SEM) and were derived from 3 biological replicate groups. Each biological replicate group consisted of 3 mixed samples from 6 fish (n=6), and each sample underwent 3 technical replicate experiments. **(A)** Contents of TC in different photoperiods; **(B)** Contents of LDL lipids in different photoperiods; **(C)** Contents of TG in different photoperiods; **(D)** Contents of NEFA in different photoperiods; Different superscript letters indicate significant differences among groups (*P*< 0.05), while the same letters indicate no significant difference.

The contents of TC, LDL, TG, and NEFA in serum were used as indicators for evaluating blood lipid concentrations. The results are shown in **Figures 3**, **4**. Blood lipid levels decreased in females and males as the photoperiod increased. The contents of TC, LDL, TG and NEFA were in the following order: 10L:14D > 12L:12D > 14L:10D > 16L:8D in females and males. The contents of TC, LDL, TG and NEFA were the highest at 10L:14D and the lowest at 16L:8D in females and males (*P*< 0.01).

### 3.4 Gene expression

**Figure 5:**
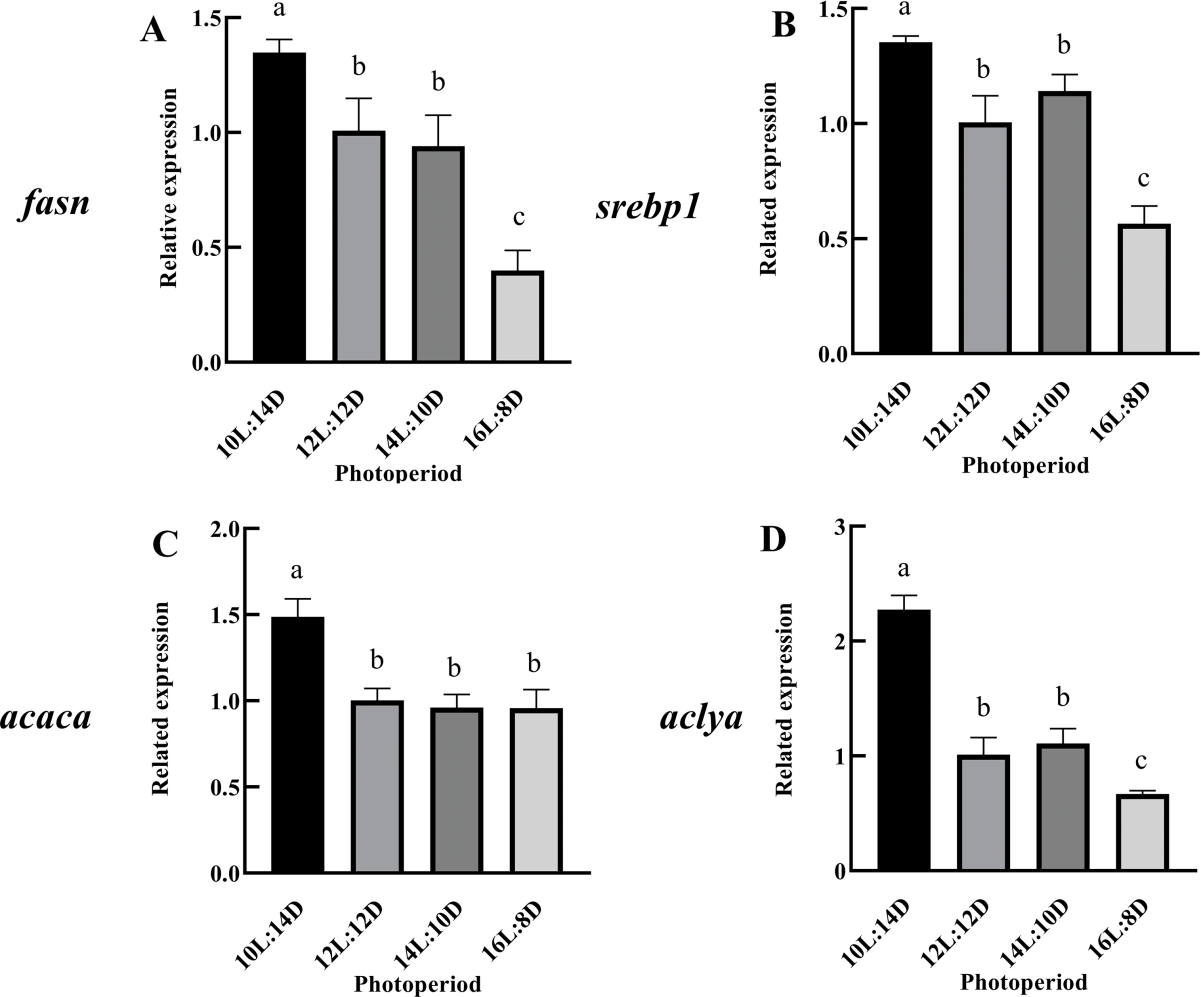
Expression levels of lipid synthesis genes in female tilapia during different photoperiods. A total of 4 photoperiod control groups were established. The values for each group are presented as the mean ± standard error of the mean (SEM) and were derived from 3 replicate sample groups (1 individual sample per replicate; n=1), with 3 technical replicates performed. **(A)** Different photoperiods expression levels of *fasn* gene; **(B)** Different photoperiods expression levels of *srebp1* gene; **(C)** Different photoperiods expression levels of *acaca* gene; **(D)** Different photoperiods expression levels of *aclya* gene; Different superscript letters indicate significant differences among groups (*P*< 0.05), while the same letters indicate no significant difference.

**Figure 6:**
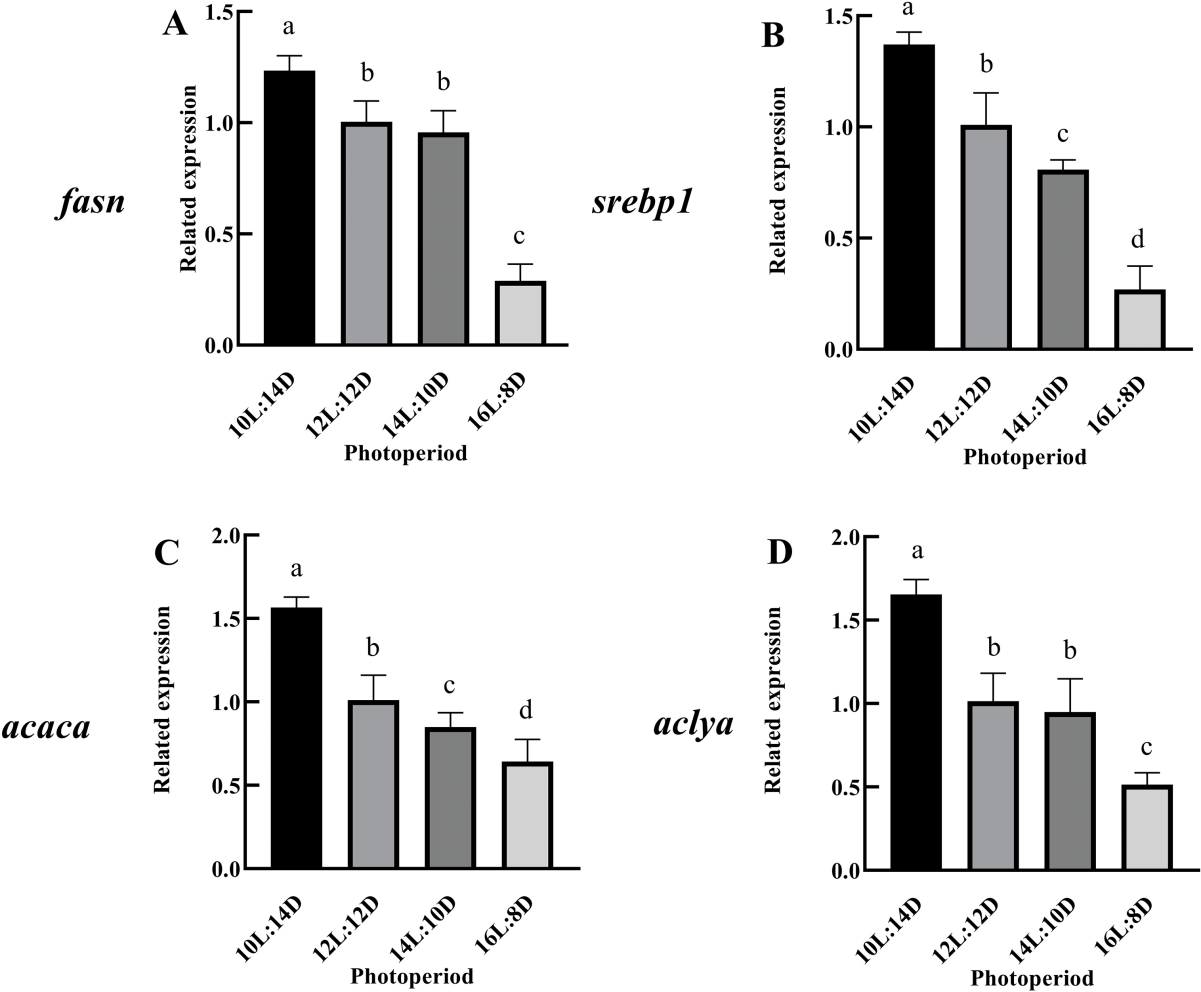
Expression levels of lipid synthesis genes in different photoperiods in male tilapia. A total of 4 photoperiod control groups were established. The values for each group are presented as the mean ± standard error of the mean (SEM) and were derived from 3 replicate sample groups (1 individual sample per replicate; n=1), with 3 technical replicates performed. **(A)** Different photoperiod expression levels of the *fasn* gene; **(B)** Different photoperiods expression levels of *srebp1* gene; **(C)** Different photoperiods expression levels of *acaca* gene; **(D)** Different photoperiods expression levels of *aclya* gene; Different superscript letters indicate significant differences among groups (*P*< 0.05), while the same letters indicate no significant difference.

The expression levels of *fasn*, *srebp1*, *acaca*, and *aclya* in the livers of female and male fish are shown in **Figures 5**, **6**. The *fasn* gene expression levels were consistent in females and males: 10L:14D > 12L:12D > 14L:10D > 16L:8D. The *srebp1* gene expression levels were as follows: 10L:14D > 14L:10D > 12L:12D > 16L:8D (*P*<0.01) in females and 10L:14D > 12L:12D > 14L:10D > 16L:8D in males (*P*< 0.01). The *acaca* gene expression levels were as follows: in females, the 10L:14D group exhibited the highest expression, with no significant differences among the remaining groups; in males, the expression levels followed the order: 10L:14D > 12L:12D > 14L:10D > 16L:8D. The *aclya* gene expression levels were as follows: 10L:14D > 14L:10D > 12L:12D > 16L:8D in females and 10L:14D > 12L:12D > 14L:10D > 16L:8D in males. The *srebp1*, *acaca*, and *aclya* gene expression in females did not show simple monotonic downregulation with increasing photoperiod, as the 14L:10D group exhibited higher expression than the 12L:12D group, indicating a non-linear response to photoperiod extension.

### 3.5 Fatty acid composition of the liver

The fatty acid composition of the liver not only constitutes the central substrate of energy metabolism but also plays important roles in cell structure, signal transduction, metabolic regulation, etc. A total of 32 fatty acids were detected among the 38 common fatty acids in this study. The fatty acids whose contents were greater than 1% were statistically analyzed, and the results are presented in **Tables 4**, **5**. The lowest total saturated fatty acid (SFA) contents were detected in females and males (*P*<0.01), at 28.07% and 29.18%, respectively, in the 16L:8D group compared with the 10L:14D, 12L:12D, and 14L:10D groups, with significant differences in total SFA content among all groups. In the 16L:8D group, the total polyunsaturated fatty acid (PUFA) contents were the highest in females and males, at 39.86% and 41.81%, respectively (*P*< 0.01), with significant differences in total PUFA content among all groups. The highest total monounsaturated fatty acid (MUFA) content in females was 34.64% at 14L:10D, while the highest total MUFA content in males was 32.33% at 12L:12D, with significant differences in total MUFA content among all groups. Among the essential fatty acids, the contents of C18:2n6c and C18:3n3 were both highest in the 16L:8D group.

**Table 4:** Fatty acid profile of female liver.

Fatty acid	10L:14D(%)	12L:12D(%)	14L:10D(%)	16L:8D(%)
C14:0	1.532 ± 0.015^b^	1.303 ± 0.008^c^	1.813 ± 0.004^a^	1.536 ± 0.001^b^
C16:0	20.549 ± 0.031^b^	19.364 ± 0.084^c^	21.277 ± 0.017^a^	19.063 ± 0.027^d^
C18:0	8.685 ± 0.014^a^	7.899 ± 0.026^b^	6.59 ± 0.010^c^	6.389 ± 0.003^d^
SFA	32.900 ± 0.030^a^	30.630 ± 0.087^c^	31.111 ± 0.040^b^	28.065 ± 0.019^d^
C16:1	2.964 ± 0.060^b^	2.748 ± 0.021^c^	3.911 ± 0.010^a^	2.760 ± 0.007^c^
C18:1n9c	26.667 ± 0.063^c^	26.923 ± 0.062^b^	29.074 ± 0.014^a^	25.027 ± 0.006^d^
C20:1	1.380 ± 0.040^a^	1.156 ± 0.014^b^	1.116 ± 0.015^b^	0.978 ± 0.005^c^
MUFA	32.199 ± 0.044^b^	31.687 ± 0.061^c^	34.641 ± 0.020^a^	29.672 ± 0.004^d^
C18:2n6c	18.771 ± 0.014^d^	22.433 ± 0.021^c^	23.422 ± 0.007^b^	24.863 ± 0.005^a^
C18:3n6	1.014 ± 0.024^c^	1.346 ± 0.025^a^	1.200 ± 0.005^b^	1.213 ± 0.007^b^
C18:3n3	1.276 ± 0.004^d^	1.463 ± 0.041^c^	1.795 ± 0.020^b^	1.885 ± 0.009^a^
C20:2	1.323 ± 0.032^b^	1.523 ± 0.040^a^	1.085 ± 0.003^c^	1.295 ± 0.009^b^
C20:3n6	1.145 ± 0.010^a^	1.174 ± 0.007^a^	0.916 ± 0.002^c^	1.044 ± 0.006^b^
C20:4n6	3.718 ± 0.023^a^	3.116 ± 0.013^b^	1.934 ± 0.034^c^	3.098 ± 0.008^b^
C22:6n3	6.714 ± 0.037^a^	5.794 ± 0.038^b^	3.249 ± 0.003^d^	5.600 ± 0.014^c^
PUFA	34.902 ± 0.054^c^	37.684 ± 0.137^b^	34.248 ± 0.022^d^	39.863 ± 0.020^a^

The value for each group is presented as mean ± standard error of the mean (SEM). It is expressed as a percentage of the total fatty acid content in the liver (%). The sample size for each group is 3 (n=3). Different superscript letters indicate significant differences among groups (*P*< 0.05), while the same letters indicate no significant difference. These letters indicated groups difference in the upper right corner of data.

**Table 5:** Fatty acid profile of male liver.

Fatty acid	10L:14D(%)	12L:12D(%)	14L:10D(%)	16L:8D(%)
C14:0	1.331 ± 0.003^d^	2.099 ± 0.007^a^	1.404 ± 0.003^c^	1.490 ± 0.001^b^
C16:0	18.837 ± 0.016^c^	19.662 ± 0.029^a^	19.523 ± 0.013^b^	18.680 ± 0.026^d^
C18:0	8.607 ± 0.011^a^	8.463 ± 0.010^b^	8.013 ± 0.028^c^	6.957 ± 0.011^d^
SFA	31.370 ± 0.004^b^	32.009 ± 0.012^a^	31.166 ± 0.023^c^	29.183 ± 0.038^d^
C16:1	2.114 ± 0.030^c^	2.564 ± 0.013^a^	2.351 ± 0.011^b^	2.366 ± 0.004^b^
C18:1n9c	24.146 ± 0.040^d^	27.812 ± 0.031^a^	24.437 ± 0.010^c^	24.875 ± 0.041^b^
C20:1	1.132 ± 0.024^c^	1.274 ± 0.004^a^	1.172 ± 0.006^c^	1.085 ± 0.011^b^
MUFA	28.294 ± 0.039^d^	32.331 ± 0.021^a^	28.700 ± 0.023^c^	29.008 ± 0.050^b^
C18:2n6c	21.621 ± 0.018^c^	21.110 ± 0.029^d^	23.245 ± 0.015^b^	26.022 ± 0.044^a^
C18:3n6	1.038 ± 0.025^a^	0.825 ± 0.002^b^	0.742 ± 0.007^c^	0.876 ± 0.009^b^
C18:3n3	1.369 ± 0.027^c^	1.380 ± 0.011^c^	1.649 ± 0.016^b^	1.888 ± 0.006^a^
C20:2	1.689 ± 0.009^a^	1.552 ± 0.026^a^	1.630 ± 0.019^a^	1.604 ± 0.006^a^
C20:3n6	1.217 ± 0.002^a^	1.031 ± 0.019^b^	1.027 ± 0.003^b^	1.026 ± 0.003^b^
C20:4n6	4.040 ± 0.007^a^	2.684 ± 0.014^d^	3.160 ± 0.025^b^	2.831 ± 0.020^c^
C22:6n3	8.560 ± 0.023^a^	6.424 ± 0.032^d^	7.861 ± 0.007^b^	6.789 ± 0.026^c^
PUFA	40.336 ± 0.040^b^	35.660 ± 0.009^d^	40.139 ± 0.045^c^	41.810 ± 0.018^a^

The value for each group is presented as mean ± standard error of the mean (SEM). It is expressed as a percentage of the total fatty acid content in the liver (%). The sample size for each group is 3 (n=3). Different superscript letters indicate significant differences among groups (*P*< 0.05), while the same letters indicate no significant difference. These letters indicated groups difference in the upper right corner of data.

### 3.6 Lipidomic results

**Figure 7:**
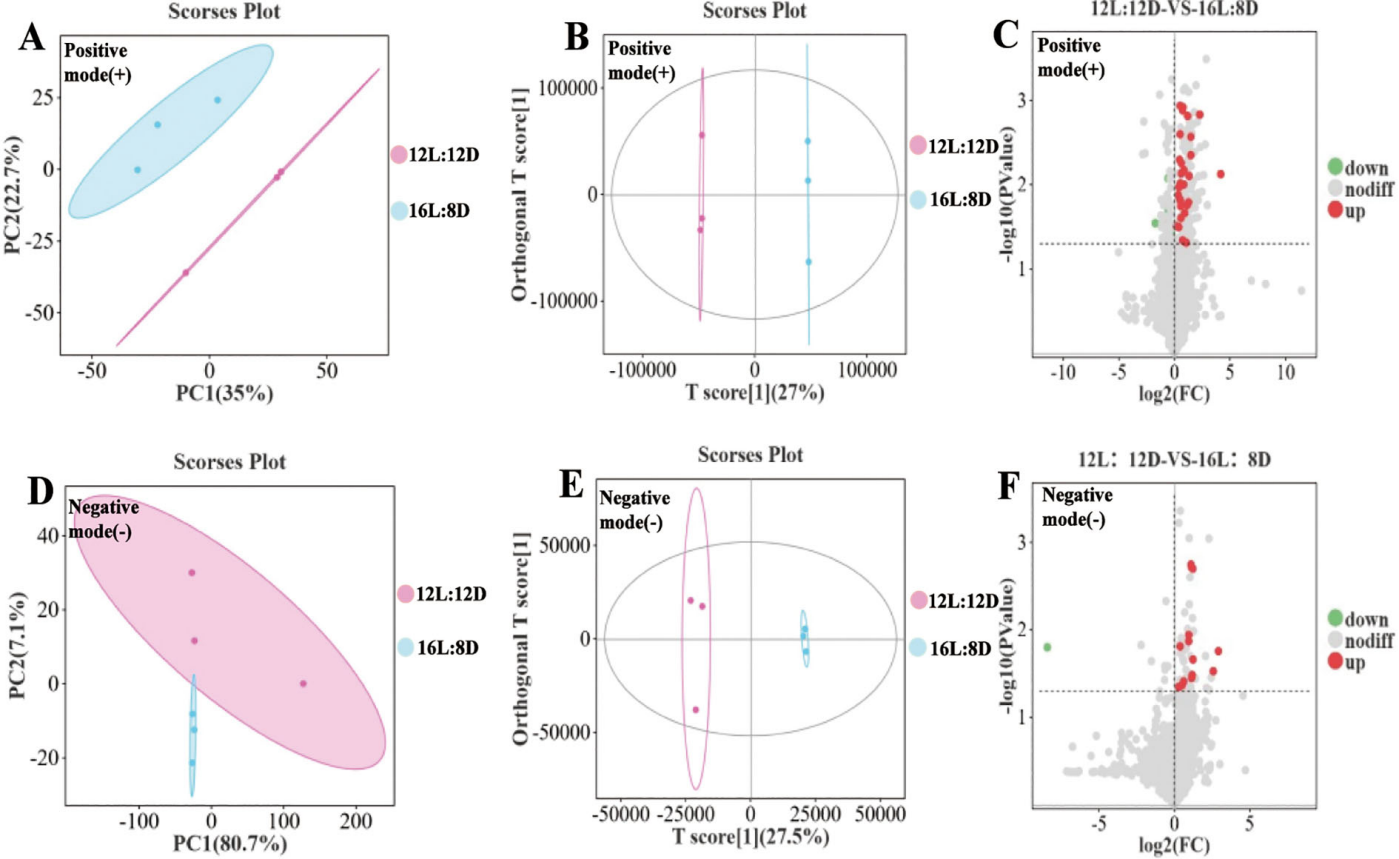
Bioinformatics analysis of differential lipid metabolites. **(A)** PCA plot in positive mode, **(B)** OPLS-DA plot in positive mode, **(C)** volcano plot in positive mode, **(D)** PCA plot in negative mode, **(E)** OPLS-DA plot in negative mode, **(F)** volcano plot in negative mode. In the experimental group (16L:8D) vs the control group (12L:12D), differentially abundant metabolites were screened based on the criteria of a VIP ≥ 1 and *P*< 0.05. There were 3 pooled samples per group (each pool contained 6 fish), with n = 3.

**Figure 8:**
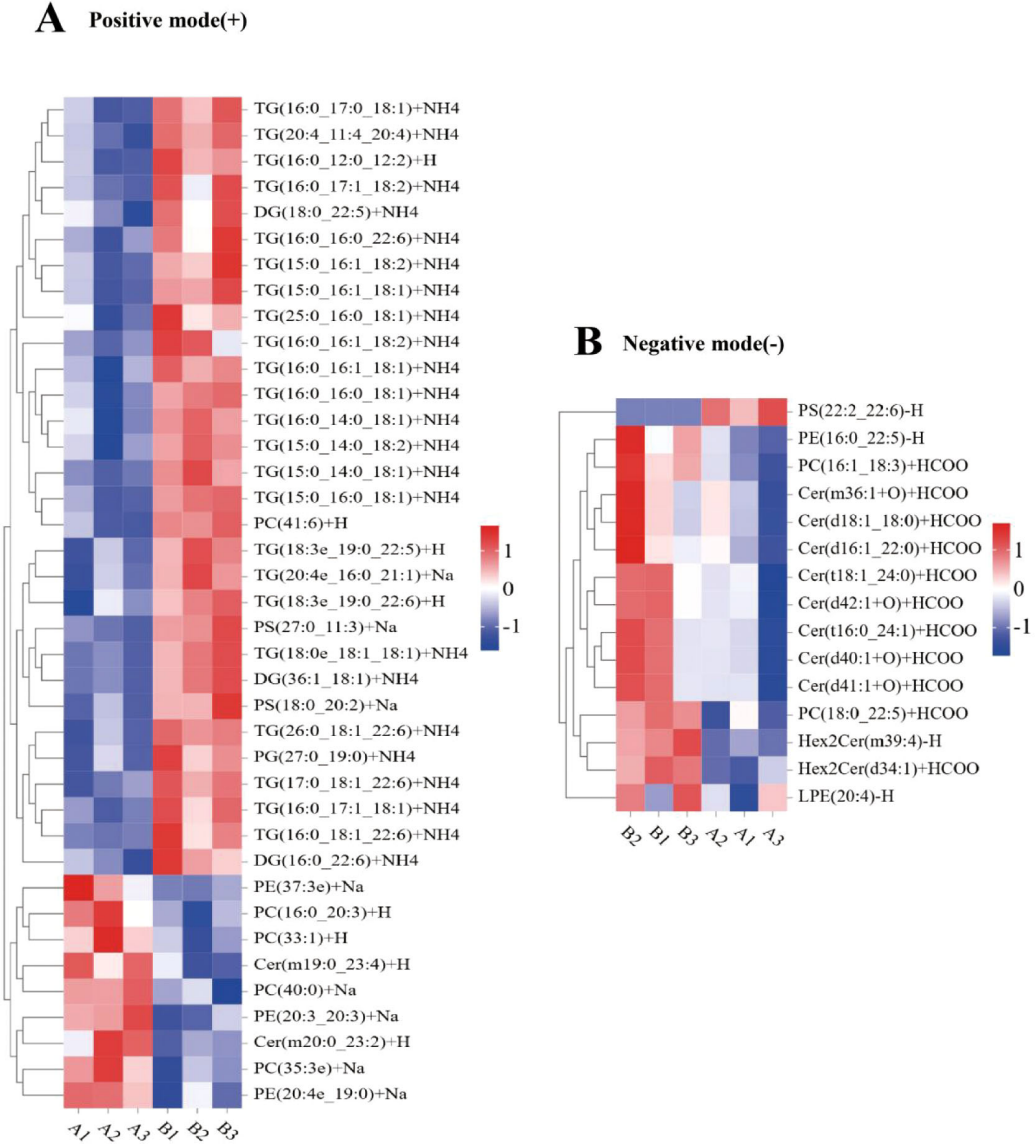
Clustered heatmaps of differential lipid metabolites. Darker red color indicates higher relative content; darker blue color indicates lower relative content. **(A)** Clustering heatmap of differential lipid metabolites in positive mode. **(B)** Clustering heatmap of differential lipid metabolites in negative mode. There were 3 pooled samples per group (each pool contained 6 fish), with n = 3.

The results of the bioinformatics analysis of the differentially abundant lipid metabolites are shown in **Figures 7**, **8**. PCA score scatter plots were used to assess the differentiation between the control group (12L:12D) and the experimental group (16L:8D) (**Figures 7A, D**). The OPLS-DA model demonstrated good differentiation between the control group and the experimental group (**Figures 7B, E**). Notably, the expression of most of the metabolites increased under long photoperiods. A total of 3,399 lipid metabolites were identified between the control group (12L:12D) and the experimental group (16L:8D), among which 54 were significantly different (*P*<0.01), including 44 metabolites whose levels increased and 10 metabolites whose levels decreased (**Figures 7C, F**). A cluster heatmap of differential lipid metabolites is shown in **Figures 8A, B**. Representative increases in differentially expressed metabolites included those of diglyceride (DG) (16:0_22:6) (log2FC 1.14) and dihexosylceramide (Hex_2_Cer) (m39:4) (log2FC 2.92). The differentially expressed metabolites that were reduced included ceramide (Cer) (m20:0_23:2) (log2FC –1.72) and phosphatidylserine (PS) (22:2_22:6) (log2FC –8.45).

## 4 Discussion

Longer photoperiods promote the growth of fish such as North American pike (*Esox lucius*) (Imentai et al., 2024), zebrafish (*Danio*) (Abdollahpour et al., 2020), and tilapia (Fuentes-Silva et al., 2015). We found that a long-day photoperiod promoted the growth of tilapia in this study, which is consistent with previous findings. The number of lipid droplets can be used to visualize the degree of lipid deposition in the body (Wölk and Fedorova, 2024). Blood lipid contents (TC, LDL, TG, and NEFA) reflect the level of lipid metabolism (Matey-Hernandez et al., 2018). Observations of oil red O−stained liver sections of tilapia in this experiment revealed that under a short photoperiod (10L:14D group), lipid droplets were densely packed and widely distributed throughout the hepatocytes. In contrast, under a long photoperiod (16L:8D group), lipid droplets became sparse, with only occasional small droplets observed, and hepatocytes displayed a normal morphology with clearly stained nuclei. The blood lipid content revealed that the levels of TC, LDL, TG, and NEFA in the photoperiod (16L:8D) group were significantly lower than those in the other groups. Scholars have reported that male Menidia beryllina (*Atherinidae*) fish exposed to 9.5- and 12-h photoperiods accumulated significantly more visceral fat than those exposed to a 15-h photoperiod (Huber and Bengtson, 1999). The results of this study reveal that long photoperiods can significantly reduce fat deposition in tilapia liver. These results are consistent with previous findings.

*fasn*, *acaca*, *srebp1* and *aclya* are the key regulatory genes of the lipid synthesis pathway in animals and are the core indicators for evaluating lipid synthesis levels (Ke et al., 2024; Liu et al., 2021a). The expression levels of lipogenic genes (*fasn*, *srebp1* and *acaca*) decreased, leading to the suppression of lipid synthesis in zebrafish (Liu et al., 2021b; Zhang et al., 2026). These findings indicated that lower expression of lipid synthesis genes (*fasn*, *acaca*, and *aclya*) in leaves reduced lipid synthesis efficiency in juvenile gibel carp (Wei et al., 2019). The results of these experiments revealed that the expression levels of the tilapia *fasn*, *acaca*, *srebp1*, and *aclya* genes were significantly downregulated under a long photoperiod (16L:8D). We reported that long photoperiods decrease lipid synthesis by suppressing the expression of the *fasn*, *srebp1*, *acaca* and *aclya* genes. The *srebp1* and *aclya* gene expression in females does not show simple monotonic downregulation with increasing photoperiod. Compared with the 12L:12D group, the 14L:10D group presented greater expression, indicating a non-linear response to photoperiod extension. Since photoperiod influences various life activities of fish, such as reproduction and growth, and lipid synthesis in females is affected by factors such as gonadal development and growth, we speculate that the observed phenomenon results from the combined effects of the female sex hormone rhythm and different photoperiods on the expression of the *srebp1* and *aclya* genes. The specific mechanisms underlying this non-linear change require further investigation.

Fat is the main source of energy for the body, and fatty acids are the basic units constituting lipids (Bahtz et al., 2009). A fatty acid is a carboxylic acid with an aliphatic chain, typically containing an even number of carbons (C4-C28), and may be a straight chain or a saturated or unsaturated branched chain (Toncan et al., 2025). Based on their degree of saturation, they can be classified into SFAs, MUFAs, and PUFA (Yuan et al., 2023). Changes in fatty acid composition are closely related to the healthy growth of organisms. The results of fatty acid profiling in this study revealed that SFA levels decreased while PUFA levels increased with prolonged photoperiod in tilapia liver. Compared with those in the other groups, the contents of saturated fatty acids such as palmitic acid (PA) and stearic acid (SA) in the liver were lower in the 16L:8D group, whereas the contents of polyunsaturated fatty acids such as α-linolenic acid (ALA) and linoleic acid (LA), were higher in the 16L:8D group. ALA belongs to the PUFA family and is an important essential fatty acid that has hepatoprotective, anti-inflammatory, and blood circulation-improving functions and promotes lipid metabolism (Ogawa et al., 2023; Poudyal et al., 2012). Studies have shown that increasing the content of ALA in the body enhances hepatic immune function in tilapia (Huang et al., 2022). These findings indicated that ALA can increase the lipid metabolism capacity of the liver in juvenile Russian sturgeon (*Acipenser gueldenstaedtii*) (Wang et al., 2019). In this study, we reported that a long photoperiod promoted an increase in ALA levels, which is beneficial for the healthy growth of tilapia.

To investigate the mechanism by which photoperiod influences lipid metabolism in tilapia, this study employed lipidomics to compare hepatic lipid profiles between a long photoperiod group (16L:8D) and a control group (12L:12D). The results of the present study revealed that 54 different metabolites were identified by lipidomic analysis, of which 44 metabolites were significantly upregulated and 10 metabolites were significantly downregulated. These lipids with different metabolic levels revealed the potential role of photoperiod in regulating lipid metabolism in tilapia. Interestingly, we found that the expression level of diglyceride (DG) was significantly elevated in the 16L:8D group. DG is a glyceride that is formed by the esterification of two hydroxyl groups of a glycerol molecule with two fatty acids (Eichmann and Lass, 2015). DGs are widely distributed in various tissues and cells of animals (Mondal et al., 2024). DG is an important secondary messenger in cellular signal transduction. In response to external stimuli (such as hormones or neurotransmitters), DG activates protein kinase C (PKC) to produce corresponding biological effects, thereby participating in vital processes such as cell proliferation and cycle regulation (Sun and Zhu, 1996). Concurrently, the DG plays a crucial role in maintaining lipid metabolic homeostasis *in vivo*. On the one hand, under the action of acyl-CoA and triglyceride synthetase, DG contributes to triglyceride synthesis and fat storage. On the other hand, DG can also affect the catabolism of fatty acids under the action of fatty acid oxidase (Lewandowska et al., 2023). Feeding experiments in mice have demonstrated that DG can reduce white adipose tissue accumulation and decrease serum levels of total cholesterol, triglycerides, and low-density lipoprotein cholesterol (Cheng et al., 2025). The results indicated that the lowest number of lipid droplets and the lowest triglyceride (TG) content in blood lipids were detected in the 16L:8D group. Furthermore, lipidomic analysis revealed that the expression level of diacylglycerol (DG) in the 16L:8D group was significantly greater than that in the control group (12L:12D). Therefore, we hypothesized that DG may be an important factor in the photoperiod regulation of lipid metabolism and fat deposition in tilapia. We will subsequently conduct relevant experiments to verify its function and clarify its biological significance.

## 5 Conclusion

In this study, we investigated the mechanism by which photoperiod regulates fat deposition in tilapia liver by tissue sectioning, blood lipid testing, fatty acid profiling, and lipidomics. These results indicate that a long photoperiod (16L:8D) effectively increases the growth rate of tilapia and reduces blood lipid concentrations and hepatic fat deposition. Additionally, several candidate biomarkers associated with photoperiod-regulated lipid metabolism were screened using lipidomic approaches. These results provide new insights into the study of fish lipid metabolism and regulation and have practical guiding significance for facility-based high-efficiency tilapia farming.

## Data Availability

The original contributions presented in the study are included in the article/supplementary material. Further inquiries can be directed to the corresponding authors.

## References

[B1] Abdollahpour H. Falahatkar B. Lawrence C. (2020). The effect of photoperiod on growth and spawning performance of zebrafish, Danio rerio. Aquacult. Rep. 17, 100295. doi: 10.1016/j.aqrep.2020.100295 38826717

[B2] Bahtz J. Knorr D. Tedeschi C. Leser M. E. Valles-Pamies B. Miller R. (2009). Adsorption of octanoic acid at the water/oil interface. Colloids. Surf. B. Biointerfaces. 74, 492–497. doi: 10.1016/j.colsurfb.2009.07.041 19766464

[B3] Cheng F. Yang X. Liu X. Wang X. Li W. Han N. . (2025). Impact of different concentrations of diacylglycerol edible oil on metabolic regulation and liver function in obese mice. China Oils. Fats. 50, 88–92. doi: 10.19902/j.cnki.zgxz.1003-7969.240270

[B4] Dreier D. A. Bowden J. A. Aristizabal-Henao J. J. Denslow N. D. Martyniuk C. J. . (2020). Ecotoxico-lipidomics: An emerging concept to understand chemical-metabolic relationships in comparative fish models. Comp. Biochem. Physiol. - Part. D. Genomics Proteomics 36, 100742. doi: 10.1016/j.cbd.2020.100742 32956922 PMC7669741

[B5] Du Z. Y. (2014). Causes of fatty liver in farmed fish: a review and new perspectives. J. Fish. China. 38, 1628–1638. doi: 10.3724/SP.J.1231.2014.49374

[B6] Eichmann T. O. Lass A. (2015). DAG tales: the multiple faces of diacylglycerol—stereochemistry, metabolism, and signaling. Cell. Mol. Life Sci. 72, 3931–3952. doi: 10.1007/s00018-015-1982-3 26153463 PMC4575688

[B7] Fu X. Zou Z. Zhu J. Xiao W. Li D. Yu J. . (2021). Effects of different photoperiods on growth performance, daily rhythm of growth axis-related genes, and hormones in Nile tilapia (*Oreochromis niloticus*). Aquaculture 553, 738071. doi: 10.1016/j.aquaculture.2022.738071 38826717

[B8] Fuentes-Silva C. Soto-Zarazúa G. M. Torres-Pacheco I. Guevara-González R. G. García-Trejo J. F. Rangel A. F. . (2015). Influence of Extended Photoperiod on All Male Nile Tilapia (*Oreochromis niloticus*) Production, Differential Gene Expression and Growth Rate. Int. J. Agric. Biol. 17 (4), 785–790. doi: 10.17957/IJAB/14.0016

[B9] Fujisawa K. Takami T. Shintani H. Sasai N. Matsumoto T. Yamamoto N. . (2021). Seasonal variations in photoperiod affect hepatic metabolism of medaka (*Oryzias latipes*). FEBS Open Bio 11, 1029–1040. doi: 10.1002/2211-5463.13095 33475250 PMC8016123

[B10] Fukada H. Yabuki H. Miura C. Miura T. Kato K. (2023). Regulation of lipid metabolism by water temperature and photoperiod in yellowtail Seriola quinqueradiata. Fish. Sci. 89, 191–202. doi: 10.1007/s12562-022-01664-4 30311153

[B11] Huang X. Chen F. Guan J. Xu C. Li Y. Xie D. (2022). Beneficial effects of re-feeding high α-linolenic acid diets on the muscle quality, cold temperature and disease resistance of tilapia. Fish. Shellfish. Immunol. 126, 303–310. doi: 10.1016/j.fsi.2022.05.053 35662581

[B12] Huber M. Bengtson D. A. (1999). Effects of photoperiod and temperature on the regulation of the onset of maturation in the estuarine fish Menidia beryllina (Cope) (*Atherinidae*). J. Exp. Mar. Biol. Ecol. 240, 285–302. doi: 10.1016/S0022-0981(99)00064-7

[B13] Imentai A. Bondarenko V. Pěnka T. Policar T. (2024). Effects of weaning time, light regime, and stocking density on growth, condition, survival, and cannibalism rates in northern pike (*Esox lucius* L.) larvae and early juveniles under intensive culture. Front. Mar. Sci. 11, 1352699. doi: 10.3389/fmars.2024.1352699

[B14] Ke S. Zhang S. Liu D. Zhao T. Lou X. Cheng S. . (2024). Ectopic OR1A1 activation ameliorates hepatic lipid deposition through AMPK/SREBP-1/FASN pathway by three monoterpenes. J. Funct. Foods. 115, 106097. doi: 10.1016/j.jff.2024.106097 38826717

[B15] Kolde R. Kolde M. R. (2015). Package ‘pheatmap’. R. Package 1, 790. doi: 10.32614/CRAN.package.pheatmap

[B16] Lewandowska M. Zienkiewicz A. Feussner K. König S. Kunst L. Feussner I. (2023). Wound-induced triacylglycerol biosynthesis is jasmonoyl-isoleucin and abscisic acid independent. Plant Biol. 25, 509–517. doi: 10.1111/plb.13513 36800436

[B17] Lin H. Jia Y. Han F. Xia C. Zhao Q. Zhang J. . (2022). Toxic effects of waterborne benzylparaben on the growth, antioxidant capacity and lipid metabolism of Nile tilapia (*Oreochromis niloticus*). Aquat. Toxicol. 248, 106197. doi: 10.1016/J.AQUATOX.2022.106197 35623196

[B18] Liu N. Ji Y. Yang Y. Jia H. Si X. Jiang D. . (2021a). Impact of dietary crude protein level on hepatic lipid metabolism in weaned female piglets. Animals 11, 1829. doi: 10.3390/ani11061829 34207398 PMC8235084

[B19] Liu Y. H. Guo H. Y. Liu B. S. Zhu T. F. Xian L. Zhang N. . (2026). Lipidomic profiling of dechorionated fertilized eggs and egg chorion in three tropical marine fish species: Insights into reproductive physiology and nutrition. Biology 15, 172. doi: 10.3390/BIOLOGY15020172 41594908 PMC12837247

[B20] Liu Y.-S. Yuan M.-H. Zhang C.-Y. Liu H.-M. Liu J.-R. Wei A.-L. . (2021b). Puerariae lobatae radix flavonoids and puerarin alleviate alcoholic liver injury in zebrafish by regulating alcohol and lipid metabolism. Biomed. Pharmacot. 134, 111121. doi: 10.1016/j.biopha.2020.111121 33341668

[B21] Livak K. J. Schmittgen T. D. (2001). Analysis of relative gene expression data using real-time quantitative PCR and the 2^-ΔΔCT^ method. Methods 25, 402–408. doi: 10.1006/meth.2001.1262 11846609

[B22] Lucon-Xiccato T. Montalbano G. Frigato E. Loosli F. Foulkes N. S. Bertolucci C. (2022). Medaka as a model for seasonal plasticity: Photoperiod-mediated changes in behaviour, cognition, and hormones. Horm. Behav. 145, 105244. doi: 10.1016/j.yhbeh.2022.105244 35988451

[B23] Malambugi A. Yu Z. Zhu W. Wang L. Song F. Limbu S. M. (2020). Effects of photoperiod on growth performance and melanogenesis pathway for skin pigmentation of Malaysian red tilapia. Aquac. Res. 51, 1824–1833. doi: 10.1111/are.14531 40046247

[B24] Malinovskyi O. Rahimnejad S. Stejskal V. Boňko D. Stará A. Velíšek J. . (2022). Effects of different photoperiods on growth performance and health status of largemouth bass (*Micropterus salmoides*) juveniles. Aquaculture 548, 737631. doi: 10.1016/j.aquaculture.2021.737631 38826717

[B25] Matey-Hernandez M. L. Williams F. M. K. Potter T. Valdes A. M. Spector T. D. Menni C. (2018). Genetic and microbiome influence on lipid metabolism and dyslipidemia. Physiol. Genomics 50, 117–126. doi: 10.1152/physiolgenomics.00053.2017 29341867 PMC5867613

[B26] Mondal S. Pal B. Sankaranarayanan R. (2024). Diacylglycerol metabolism and homeostasis in fungal physiology. FEMS Yeast. Res. 24, foae036. doi: 10.1093/femsyr/foae036 39611318 PMC11631473

[B27] Moran D. Softley R. Warrant E. J. (2014). Eyeless Mexican cavefish save energy by eliminating the circadian rhythm in metabolism. PloS One 9, e107877. doi: 10.1371/journal.pone.0107877 25251018 PMC4176717

[B28] Mortimer T. Smith J. G. Muñoz-Cánoves P. Benitah S. A. (2025). Circadian clock communication during homeostasis and ageing. Nat. Rev. Mol. Cell Biol. 26 (4), 314–331. doi: 10.1038/s41580-024-00802-3 39753699

[B29] Navarro-Masip E. Caron A. Mulero M. Arola L. Aragonès G. (2023). Photoperiodic remodeling of adiposity and energy metabolism in non-human mammals. Int. J. Mol. Sci. 24, 1008. doi: 10.3390/ijms24021008 36674520 PMC9865556

[B30] Nisembaum L. G. Martin P. Lecomte F. Falcón J. (2021). Melatonin and osmoregulation in fish: A focus on Atlantic salmon Salmo salar smoltification. J. Neuroendocrinol. 33, e12955. doi: 10.1111/jne.12955 33769643

[B31] Ogawa T. Sawane K. Ookoshi K. Kawashima R. (2023). Supplementation with flaxseed oil rich in alpha-linolenic acid improves verbal fluency in healthy older adults. Nutrients 15, 1499. doi: 10.3390/nu15061499 36986229 PMC10056498

[B32] Poudyal H. Panchal S. K. Waanders J. Ward L. Brown L. (2012). Lipid redistribution by α-linolenic acid-rich chia seed inhibits stearoyl-CoA desaturase-1 and induces cardiac and hepatic protection in diet-induced obese rats. J. Nutr. Biochem. 23, 153–162. doi: 10.1016/j.jnutbio.2010.11.011 21429727

[B33] Qiang J. He J. Zhu J.-H. Tao Y.-F. Bao J.-W. Yan Y. . (2021). Optimal combination of temperature and photoperiod for sex steroid hormone secretion and egg development of *Oreochromis niloticus* as determined by response surface methodology. J. Therm. Biol. 97, 102889. doi: 10.1016/j.jtherbio.2021.102889 33863448

[B34] Sun J. Zhu P. (1996). The mechanism of diacylglycerol in regulating cellular functions. Prog. Biochem. Biophys. 23, 40–42.

[B35] Tang Z. Zhou Y. Xiao J. Zhong H. Miao W. Guo Z. . (2019). Transcriptome analysis of ovary development in Nile tilapia under different photoperiod regimes. Front. Genet. 10, 894. doi: 10.3389/fgene.2019.00894 31608122 PMC6761324

[B36] Thevenot E. A. (2016). ropls: PCA, PLS(-DA) and OPLS(-DA) for multivariate analysis and feature selection of omics data. R. Package 1. doi: 10.18129/B9.bioc.ropls

[B37] Toncan F. Raj R. R. Lee M. J. (2025). Dynamics of Fatty Acid Composition in Lipids and Their Distinct Roles in Cardiometabolic Health. Biomolecules 15, 696. doi: 10.3390/biom15050696 40427589 PMC12110056

[B38] Wang X. Li Q. Wang J. Li E. Qin J. G. Chen L. (2019). Effects of dietary alpha-linolenic acids on growth performance, lipid metabolism and antioxidant responses of juvenile Russian sturgeon Acipenser gueldenstaedtii. Aquacult. Nutr. 25, 184–193. doi: 10.1111/anu.12842 40046247

[B39] Wang J. Liang X. Gao H. T. Yang H. C. Li J. C. Xiao T. . (2025). Analysis of lipid metabolic characteristics of five indigenous fish in Jinsha River based on LC-MS. Southwest. China J. Agric. Sci. 38 (12), 2678–2691. doi: 10.16213/j.cnki.scjas.2025.12.015

[B40] Wang W. Su S. Dong P. Feng W. Li J. Zhang C. . (2023). Effects of seasonal photoperiod on growth, lipid metabolism, and antioxidant response in the Huanghe carp (*Cyprinus carpio haematopterus*). Fishes 8, 595. doi: 10.3390/fishes8120595 30654563

[B41] Warnes G. R. Bolker B. Lumley T. Johnson R. C. Schwartz M. Rogers J. . (2024). gmodels: various R programming tools for model fitting (R package version 2.19.1). (Vienna, Austria: R Foundation for Statistical Computing). Available online at: https://CRAN.R-project.org/package=gmodels (Accessed June 4, 2026).

[B42] Watterson A. Douglas P. M. (2022). Intracellular lipid surveillance: Modulating protein dynamics through lipid sensing. Clin. Transl. Med. 12, e1147. doi: 10.1002/ctm2.1147 36536483 PMC9763532

[B43] Wei H. Cai W.-J. Liu H.-K. Han D. Zhu X.-M. Yang Y.-X. . (2019). Effects of photoperiod on growth, lipid metabolism and oxidative stress of juvenile gibel carp (*Carassius auratus*). J. Photochem. Photobiol. B. Biol. 198, 111552. doi: 10.1016/j.jphotobiol.2019.111552 31382089

[B44] Wölk M. Fedorova M. (2024). The lipid droplet lipidome. FEBS Lett. 598, 1215–1225. doi: 10.1002/1873-3468.14874 38604996

[B45] Wood S. H. Hindle M. M. Mizoro Y. Cheng Y. Saer B. R. C. Miedzinska K. . (2020). Circadian clock mechanism driving mammalian photoperiodism. Nat. Commun. 11, 4291. doi: 10.1038/s41467-020-18061-z 32855407 PMC7453030

[B46] Xiong W. Guo C. Gozlan R. E. Liu J. (2023). Tilapia introduction in China: Economic boom in aquaculture versus ecological threats to ecosystems. Rev. Aquacult. 15, 179–197. doi: 10.1111/raq.12710 40046247

[B47] Yuan C. Wang J. Lu W. (2023). Regulation of semen quality by fatty acids in diets, extender, and semen. Front. Vet. Sci. 10, 1119153. doi: 10.3389/fvets.2023.1119153 37180054 PMC10174315

[B48] Zhang G. Yang Y. Huang Z. Zheng S. S. Feng X. Y. Li J. . (2025). Effects of dietary GABA levels on growth, muscle quality, and liver lipid profile: Insights from lipidomics in juvenile yellowfin seabream Acanthopagrus latus. Foods 14, 2761. doi: 10.3390/foods14162761 40870675 PMC12385394

[B49] Zhang Z. Zhou K. Chen Y. Xie K. Zhao M. Zhao S. . (2026). Non-additive effects of norethisterone and levonorgestrel mixtures on lipid metabolism at environmentally relevant concentrations. Aquat. Toxicol. 291, 107686. doi: 10.1016/j.aquatox.2025.107686 41443170

